# Supplementary material for: Vγ9+Vδ2+ T cell control of Listeria monocytogenes growth in infected epithelial cells requires butyrophilin 3A genes
Source: Sci Rep. 2023 Oct 30;13:18651. doi: 10.1038/s41598-023-45587-1 (PMC10616279; doi:10.1038/s41598-023-45587-1)
Supplement: Supplementary file 1 — Supplementary Figures. [file 41598_2023_45587_MOESM1_ESM.pdf]

Vγ9+Vδ2+ T cell control of *Listeria monocytogenes* growth in infected epithelial cells requires butyrophilin 3A genes

Katrin Fischer[1,2, #], Michaela Bradlerova[1,2, #], Thomas Decker[1,2,†*] and Verena Supper[3,†]

[1]Max Perutz Labs, Vienna Biocenter Campus (VBC), Vienna, Austria

[2] Department of Microbiology, Immunobiology and Genetics, Center for Molecular Biology, University of Vienna, Vienna, Austria

[3]Boehringer Ingelheim RCV GmbH & Co KG, Vienna, Austria

[#] These authors contributed equally and share first authorship

[†] These authors contributed equally and share last authorship

*** Correspondence:**
Corresponding Author
Thomas.decker@univie.ac.at

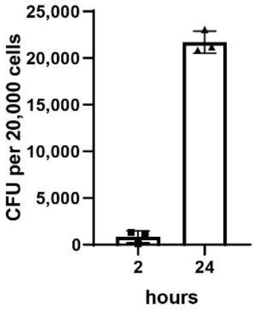

Supplementary Figure S1

Colony forming unit assay was performed using *L. monocytogenes* infected wildtype RKO cells. Cells were lysed 2 and 24 hours post infection and intracellular bacterial numbers determined. Values represent the mean and standard deviation of 3 biological replicates.

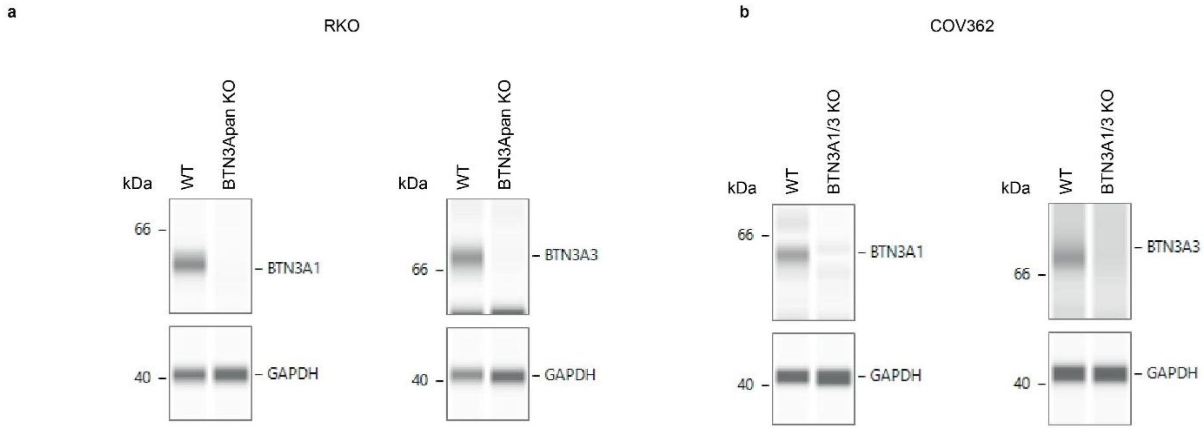

Supplementary Figure S2

BTN3A1 and BTN3A3 protein levels of **(a)** WT RKO cells and RKO cells with knockout of all 3 BTN3A genes (BTN3Apan KO). **b)** BTN3A1 and BTN3A3 protein levels of WT COV362 cells and COV362 cells with BTN3A1/3 double knockout (BTN3A1/3 KO) using the automated Western blot system WES. One representative WES image is shown **(a, b)**.

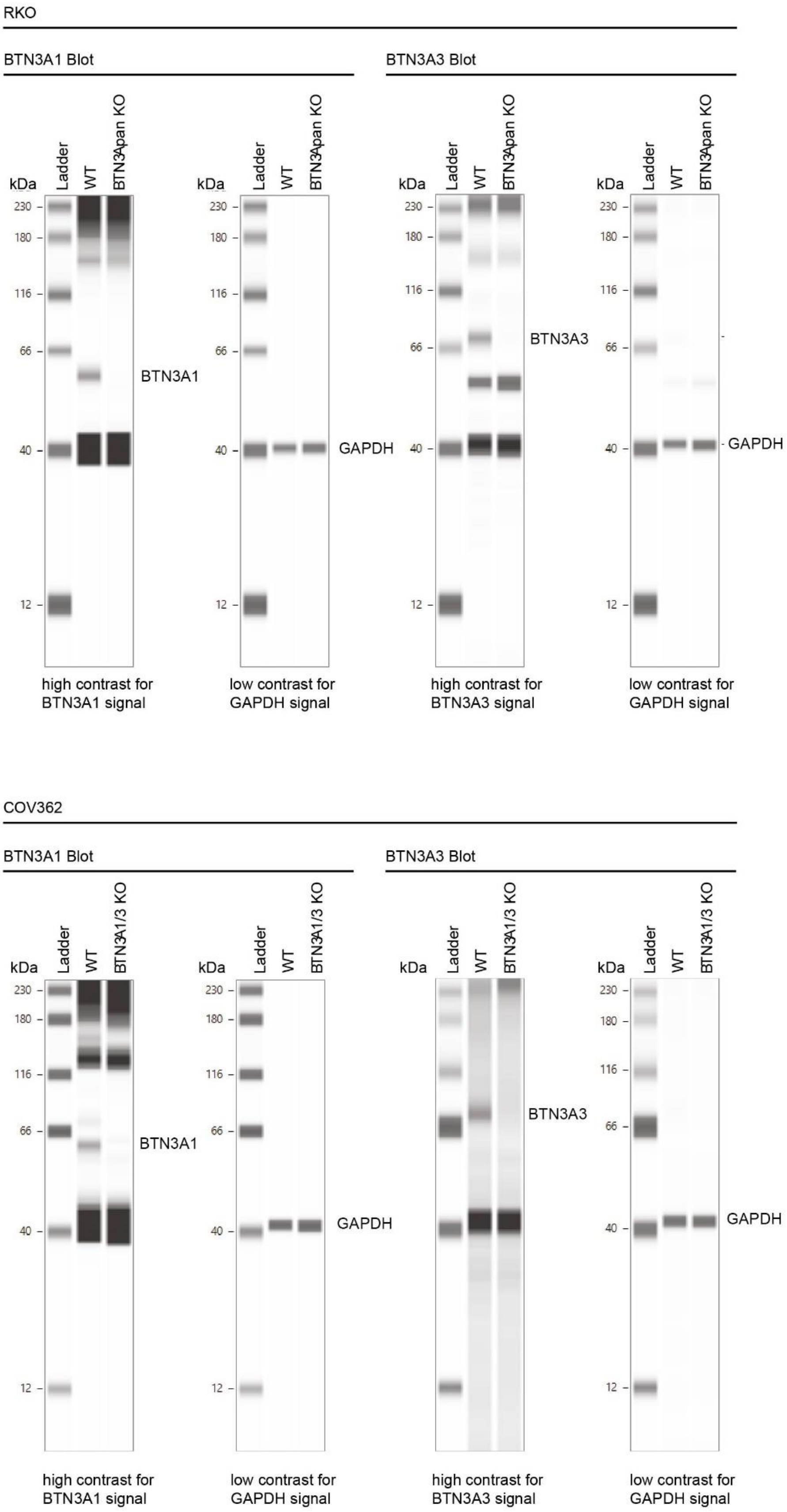
RKO
BTN3A1 Blot
BTN3A3 Blot
kDa
Ladder
WT
BTN3Apan KO
230
180
116
66
40
12
BTN3A1
GAPDH
BTN3A3
high contrast for BTN3A1 signal
low contrast for GAPDH signal
high contrast for BTN3A3 signal
low contrast for GAPDH signal
COV362
BTN3A1 Blot
BTN3A3 Blot
kDa
Ladder
WT
BTN3A1/3 KO
BTN3A1
GAPDH
BTN3A3
high contrast for BTN3A1 signal
low contrast for GAPDH signal
high contrast for BTN3A3 signal
low contrast for GAPDH signal

Supplementary Figure S3

Uncropped visualization of Supplementary Figure S2 showing the BTN3A1, BTN3A3 and GAPDH protein levels detected by WES, a capillary-based, Simple Western™ automated Western blot system. High contrast images of weaker BTN3A1 and BTN3A3 signals and low contrast images for stronger GAPDH signals are shown from RKO and COV362 WT and KO cell lysates.

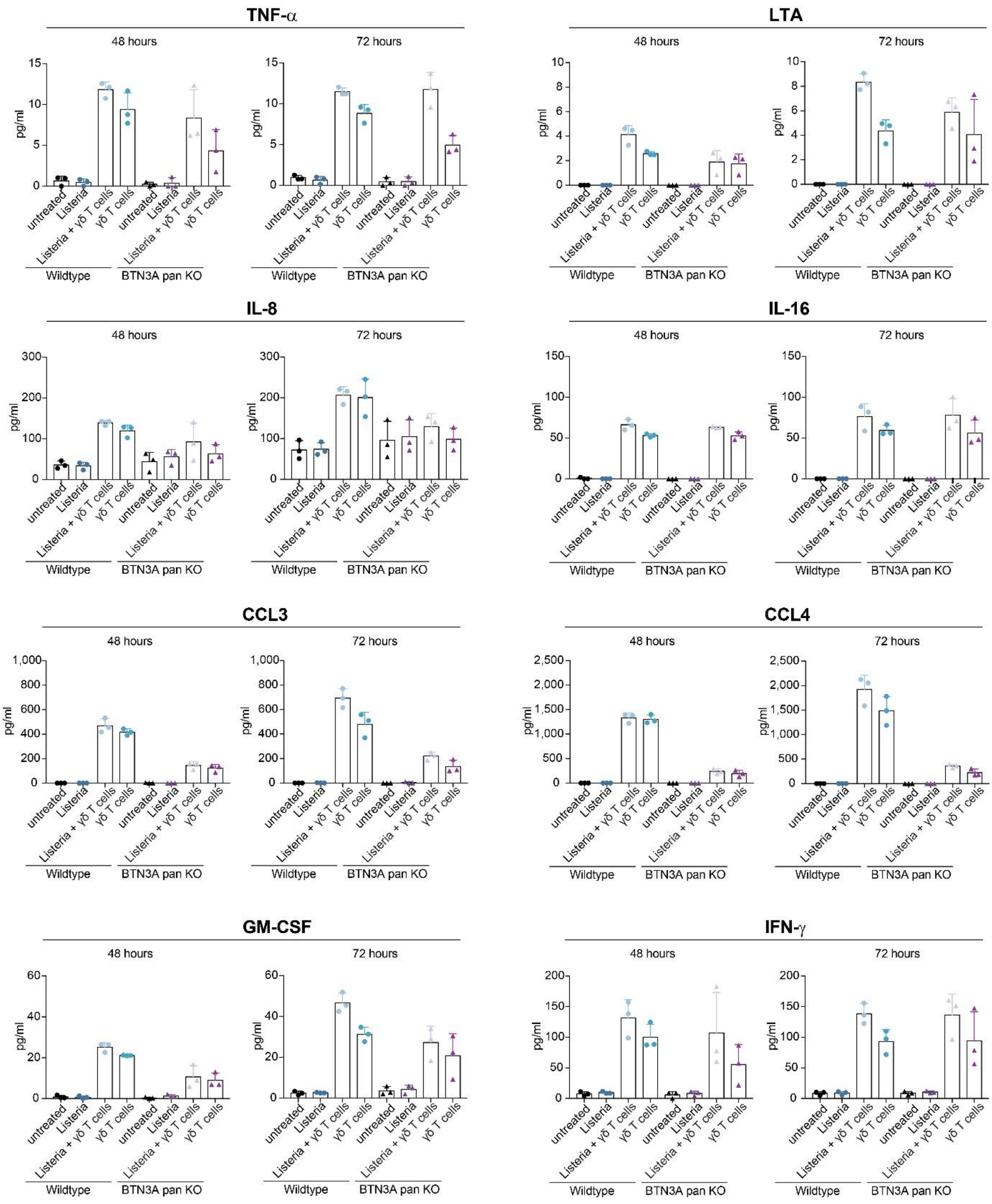

Supplementary Figure S4

Supernatants of Vγ9+Vδ2+ T cell (donor 1) co-cultures with *L. monocytogenes*-infected or uninfected wildtype and BTN3A pan knockout RKO cells were analyzed using MSD Multi-Spot-Assay. Supernatants were either taken after 48 or 72 hours. Means and standard deviations from three biological replicates are shown.

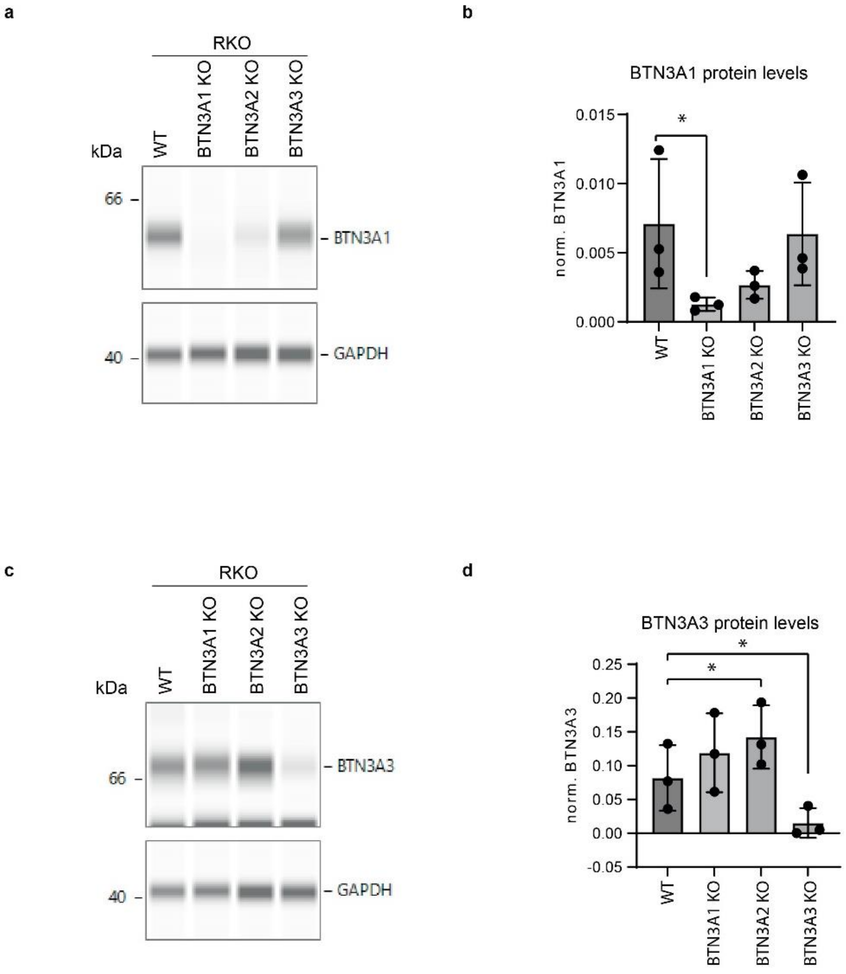

Supplementary Figure S5

BTN3A1 and BTN3A3 protein levels from single gene KO of BTN3A family members in RKO cells as determined with the automated Western blot system WES **(a-d)**. For BTN3A1 protein levels one representative WES **(a)** and a summary from 3 biological replicates of relative BTN3A1 intensity values normalized to GAPDH **(b)** are shown. For BTN3A3 also one representative WES **(c)** and a summary of 3 biological replicates of relative BTN3A1 intensity values normalized to GAPDH are shown **(d**, see also supplementary table 1**)**. Mean plus SD are depicted, and statistical significance was tested using GraphPad Prism one-way ANOVA Dunnets multiple comparison test with a single pooled variance; $*P \leq 0.05$ **(b, d)**.

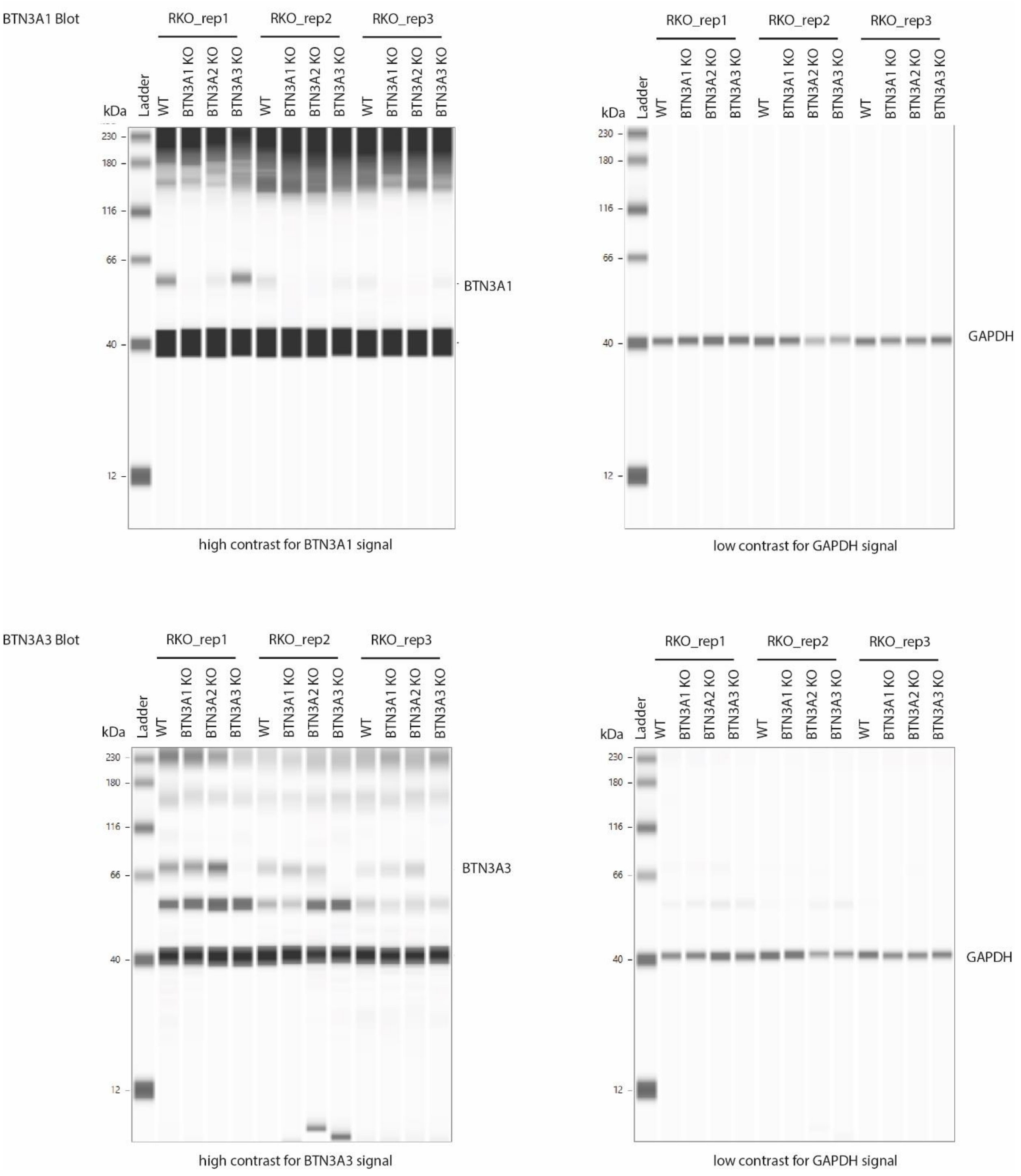

Supplementary Figure S6

Uncropped visualization of the western blots shown in Supplementary Figure S5. BTN3A1, BTN3A3 and GAPDH protein levels were detected by WES, a capillary-based, Simple Western™ automated Western blot system. High contrast images of weaker BTN3A1 and BTN3A3 signals and low contrast images for stronger GAPDH signals are shown from RKO WT and KO cell lysates.
