# Supplementary material for: Vγ9+Vδ2+ T cell control of Listeria monocytogenes growth in infected epithelial cells requires butyrophilin 3A genes
Source: Sci Rep. 2023 Oct 30;13:18651. doi: 10.1038/s41598-023-45587-1 (PMC10616279; doi:10.1038/s41598-023-45587-1)
Supplement: Supplementary file 2 — Supplementary Tables. [file 41598_2023_45587_MOESM2_ESM.docx]

**Vγ9+Vδ2+ T cell control of *Listeria monocytogenes* growth in infected epithelial cells requires butyrophilin 3A genes**

**Katrin Fischer^1,2, #^**, **Michaela Bradlerova^1,2, #^, Thomas Decker^1,2,†*^ and Verena Supper^3,†^**

^1^Max Perutz Labs, Vienna Biocenter Campus (VBC), Vienna, Austria

^2^ Department of Microbiology, Immunobiology and Genetics, Center for Molecular Biology, University of Vienna, Vienna, Austria

^3^Boehringer Ingelheim RCV GmbH & Co KG, Vienna, Austria

^#^ These authors contributed equally and share first authorship

**^†^** These authors contributed equally and share last authorship

*** Correspondence:**Corresponding Author
[Thomas.decker@univie.ac.at](mailto:Thomas.decker@univie.ac.at)

|  | Sample | BTN3A1 | GAPDH | BTN3A1 norm. to GAPDH |
| --- | --- | --- | --- | --- |
| RKO_rep1 | WT | 30,930.9 | 2,490,155.4 | 0.0124 |
|  | BTN3A1 KO | 4,827.9 | 2,714,107.0 | 0.0018 |
|  | BTN3A2 KO | 11,304.2 | 3,056,798.5 | 0.0037 |
|  | BTN3A3 KO | 31,105.5 | 2,683,209.1 | 0.0116 |
| RKO_rep2 | WT | 15,417.3 | 2,929,762.6 | 0.0053 |
|  | BTN3A1 KO | 2,129.2 | 2,645,406.5 | 0.0008 |
|  | BTN3A2 KO | 5,209.7 | 1,997,929.8 | 0.0026 |
|  | BTN3A3 KO | 7,230.1 | 2,051,032.0 | 0.0035 |
| RKO_rep3 | WT | 9,025.7 | 2,513,328.5 | 0.0036 |
|  | BTN3A1 KO | 2,748.5 | 2,243,746.8 | 0.0012 |
|  | BTN3A2 KO | 3,853.0 | 2,292,507.2 | 0.0017 |
|  | BTN3A3 KO | 8,561.4 | 2,553,466.4 | 0.0034 |

|  | RKO_rep1 | RKO_rep2 | RKO_rep3 | mean |
| --- | --- | --- | --- | --- |
| WT | 0.0124 | 0.0053 | 0.0036 | 0.0071 |
| BTN3A1 KO | 0.0018 | 0.0008 | 0.0012 | 0.0013 |
| BTN3A2 KO | 0.0037 | 0.0026 | 0.0017 | 0.0027 |
| BTN3A3 KO | 0.0116 | 0.0035 | 0.0034 | 0.0062 |

**Supplementary Table S1**

Source data generated by the automated Western blot system (WES) for BTN3A1 expression level quantification in Supplementary Figure S5b (‘BTN3A1 cartridge’). Chemiluminescent raw intensity values (AUC) of BTN3A1 and the internal control GAPDH from the BTN3A1 cartridges are shown. Raw intensity values of BTN3A1 were normalized to respective internal GAPDH control for each replicate and KO and the calculated mean, that is also illustrated in Supplementary Figure S5b is shown.

|  | Sample | BTN3A3 | GAPDH | BTN3A3 norm. to GAPDH |
| --- | --- | --- | --- | --- |
| RKO_rep1 | WT | 98,206.3 | 742,142.8 | 0.1323 |
|  | BTN3A1 KO | 143,698.3 | 806,766.8 | 0.1781 |
|  | BTN3A2 KO | 198,260.9 | 1,023,669.6 | 0.1937 |
|  | BTN3A3 KO | 3,246.8 | 892,324.5 | 0.0036 |
| RKO_rep2 | WT | 76,213.7 | 991,891.9 | 0.0768 |
|  | BTN3A1 KO | 116,459.2 | 992,119.6 | 0.1174 |
|  | BTN3A2 KO | 85,948.9 | 653,810.5 | 0.1315 |
|  | BTN3A3 KO | 0.1 | 700,158.3 | 0.0000 |
| RKO_rep3 | WT | 30,596.0 | 852,876.6 | 0.0359 |
|  | BTN3A1 KO | 44,167.6 | 719,187.8 | 0.0614 |
|  | BTN3A2 KO | 73,768.6 | 724,684.1 | 0.1018 |
|  | BTN3A3 KO | 0.0 | 778,141.2 | 0.0000 |

|  | RKO_rep1 | RKO_rep2 | RKO_rep3 | mean |
| --- | --- | --- | --- | --- |
| WT | 0.1323 | 0.0768 | 0.0359 | 0.0817 |
| BTN3A1 KO | 0.1781 | 0.1174 | 0.0614 | 0.1190 |
| BTN3A2 KO | 0.1937 | 0.1315 | 0.1018 | 0.1423 |
| BTN3A3 KO | 0.0036 | 0.0000 | 0.0000 | 0.0012 |

**Supplementary Table S2**

Source data generated by the automated Western blot system (WES) for BTN3A3 expression level quantification in Supplementary Figure S5d (‘BTN3A3 cartridge’). Chemiluminescent raw intensity values (AUC) of BTN3A3 and the internal control GAPDH from the BTN3A3 cartridges are shown. Raw intensity values of BTN3A3 values were normalized to respective internal GAPDH control for each replicate and KO and the calculated mean, that is also illustrated in Supplementary Figure S5d is shown.
